# Recommendations and guidelines for dentists using the basic erosive wear examination index (BEWE)

**DOI:** 10.1038/s41415-020-1246-y

**Published:** 2020-02-14

**Authors:** Vicente Aránguiz, Juan Sebastián Lara, M. Loreto Marró, Saoirse O’Toole, Valeria Ramírez, David Bartlett

**Affiliations:** 030631704121602477360 Facultad de Odontología, Cariology Unit, Universidad de los Andes, Colombia; 703553250160670753809 Department of Cariology, Operative Dentistry and Dental Public Health, Indiana University School of Dentistry, USA; 319873565211601546417 Prosthodontics, Faculty of Dentistry, Oral and Craniofacial Sciences, Kings College London, London, UK; 847676143514787469643 Facultad de Odontología, Cariology, Epidemiology and Public Health Unit, Universidad de los Andes, Colombia

## Abstract

This paper explains how to screen tooth wear in general practice using the Basic Erosive Wear Examination (BEWE) index. It explains how stakeholders in the UK acknowledged the convenience of the BEWE and that it could be recorded at the same time as the Basic Periodontal Examination (BPE). The article contains examples of anterior and posterior tooth wear for each BEWE score to help dentists in their evaluation.

## Key points

- The BEWE is a simple screening tool.
- THE BEWE is designed to follow a similar procedure at the BPE.
- THE BEWE should be used for every new patient examination.

## Introduction

Health Education England recently issued guidelines that recording tooth wear is aspirational rather than an essential requirement.^1^ We know that not every dentist records tooth wear, and those that do use a multitude of different methods, from indices to terms such as mild, moderate or severe. A standardised format to record tooth wear is ideal and one that is familiar can facilitate better uptake. The BEWE index (Table 1) was devised in 2008 as a screening tool for general practitioners to help with routine dental examinations.^2^ Recently in the UK, a group of stakeholders (The Royal College of General Dental Practitioners, The British Society of Dental Hygienists, GSK, Dental Protection, The Erosive Tooth Wear Foundation, King's College London and the British Dental Association) united to promote that every routine dental examination should include an assessment of erosive tooth wear.^3^

**Table 1:** BEWE Index assessment (score and description)

Score	Description
0	No erosive tooth wear
1	Initial loss of surface texture (brightness loss, opaque surface or 'frosted glass' appearance)
2	Distinct defect, hard tissue loss, less than 50% of the surface area. Dentin could be involved
3	Hard tissue loss in more than 50% of the surface area. Dentin could be involved
BEWE index assesses the damage according to the tooth affected surface regardless its depth in dentin.^2^ Sextants' cumulative assessment (maximum 18) defines the BEWE index value per assessed subject, allowing the clinical management actions according to risk.	

The group recommended that the BEWE should be performed at the same time as the BPE to save practitioners time and to utilise an already established routine and recording grid. The BEWE and the BPE use the same procedure and similar scoring system, thus can be recorded at the same time. The benefit of recording tooth wear at every clinical examination is that it is less likely that tooth wear will be missed. Ensuring it is part of every examination limits the risk of early signs not being recognised in patients and prevention not being started. A toolkit for practitioners has been produced and is available on The Erosive Tooth Wear Foundation website (www.erosivetoothwear.com), which includes free online CPD, uploads to practice-based software to enable recording of the BEWE and further information.

There is some understandable confusion regarding the application of the term 'Basic Erosive Wear Examination'. As the BEWE has evolved from a European consensus, the term tooth wear has developed a different connotation for UK dentists compared to that of our European colleagues. When the BEWE was launched, it was felt in Europe that erosion was the most important agent for tooth wear, hence there was a strong influence to refer to 'erosive tooth wear'. A large emphasis is placed on recognising that severe tooth wear rarely happens without a contributing acidic aetiology. In the UK and other countries, the term tooth wear is preferred. The BEWE gained international acceptance and it is now too late to change the terminology. The index scores changes to the surface of teeth regardless of the aetiology, so it should be used for all causes of tooth wear - including abrasion and attrition. This paper provides practical guidance to dentists on using the BEWE.

## Recommendation and guidelines

The severity of tooth wear should be evaluated in a logical and systematic way and, ideally, at the same time as the BPE at every routine clinical examination. In some dental practice software applications, this is already possible.

Ideally, the teeth should be cleaned before a clinical examination and then the buccal, occlusal and/or incisal and lingual/palatal surfaces should be assessed in each sextant under good lighting. Third permanent molars are generally excluded but should be considered if they replace a second permanent molar. Restorations that cover more than 50% of the total surface should be discarded and other surfaces in the sextant used to indicate the score. No sign of erosive tooth wear is allocated a BEWE score of zero. If there is initial loss of surface texture (brightness loss, opaque surface or 'frosted glass' appearance), a BEWE score of one is allocated. If there is a distinct wear defect with hard tissue loss but affecting less than 50% of the surface area, it is a BEWE score of two. If the loss affects more than 50% of the surface, it is a grade three. These are explained in more detail for each surface below. When doubt occurs between different scores, the lesser BEWE score should be used. Each sextant is scored based upon the worst affected surface in that sextant and is recorded in the same grid format as the BPE. Again, similar to the BPE, single remaining teeth in each sextant are added to the adjacent sextant. To get an overall score for the mouth, each sextant score can be added together to give a maximum value of 18. This guides the clinical management actions alongside risk and patient factors.^2^

When using the BEWE index with children and the primary/deciduous teeth, the same protocols should be used. The same simple grid format can be used, with the sextants divided into anteriors and posteriors. During the mixed dentition, the assessment should be the same but it is recognised that deciduous teeth are more prone to wear than adult and so the scoring is likely to be higher. The risk assessment should account for this and the time interval for repetition of the examination should be decreased to between 6 to 12 months in high-risk cases.

For patients over the age of 20 years, it is rare that their dentition is completely wear-free. A BEWE score of one is normal but, when in doubt, the lower score should be chosen. The distinction between grade zero and one is minor, but the most important distinction is between two and three. The BEWE is not designed to be used to assess progression. Like the BPE, it is a single assessment and is an adjunct to the clinical judgement at the time. It is not sufficiently accurate to enable progression to be assessed over time.

### The occlusal surfaces of molars and premolars

#### BEWE score 0

- No tooth wear signs on the occlusal surface
- Occlusal surfaces with no signs of tooth wear around a restoration interface or cusp
- Enamel developmental defects, opacities, fluorosis and amelogenesis are scored 0 when they do not involve changes to the shape of teeth due to wear.

**Fig. 1:**
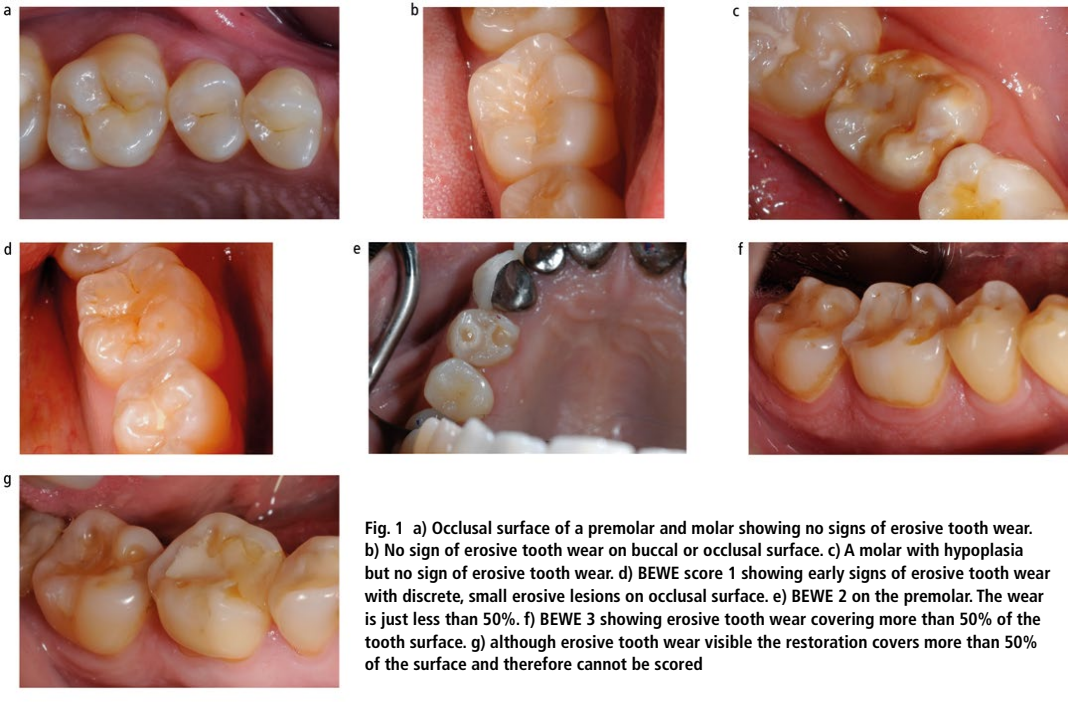
a) Occlusal surface of a premolar and molar showing no signs of erosive tooth wear. b) No sign of erosive tooth wear on buccal or occlusal surface. c) A molar with hypoplasia but no sign of erosive tooth wear. d) BEWE score 1 showing early signs of erosive tooth wear with discrete, small erosive lesions on occlusal surface. e) BEWE 2 on the premolar. The wear is just less than 50%. f) BEWE 3 showing erosive tooth wear covering more than 50% of the tooth surface. g) although erosive tooth wear visible the restoration covers more than 50% of the surface and therefore cannot be scored

These are presented in Figures 1a, 1b, and 1c.

#### BEWE Score 1

- First signs of tooth wear with rounding of the cusps and grooves
- Concavities on cusps (cupping) with diameter ≤0.5 mm (use the WHO probe to assess its diameter since its tip has a greater size). More than one cupping can be found on a single surface.

These are presented in Figure 1d

#### BEWE Score 2

- Distinct defect with tooth wear less than 50% of the whole surface area. Dentine is often involved
- Concave wear on cusps (cupping) with diameter ≥0.5 mm (it is possible to use the WHO probe to assess its diameter since its tip fits perfectly into the defect) and overall <50%
- On restored teeth: tooth wear is not related to the restoration marginal interface.

These are presented in Figure 1e.

#### BEWE Score 3

- Hard tissue loss signs for more than 50% of the surface area and dentine is often involved
- Concavities merging (cupping) can be visible but the total or near-total loss of the occlusal surface covers more than 50%
- On restored teeth: if tooth wear is seen adjacent to a proud restoration and affects >50% of the surface, it is a BEWE 3; however, if the restoration covers more than 50% of the surface, it cannot be scored.

These are presented in Figures 1f and 1g.

### The palatal/lingual and buccal surfaces of anterior teeth

When evaluating the surface in relation to the crown, consider the concept of clinical crown height as the area from the gingival margin (regardless of periodontal status) to the incisal/occlusal edge.

#### BEWE Score 0

- No tooth wear signs on the buccal/palatal/lingual surface
- Enamel developmental defects, opacities, fluorosis, amelogenesis and others are scored 0 when they do not involve changes to the shape of teeth due to wear
- Anatomical defects can be present but, provided they show no signs of wear, they can be scored as 0.

**Fig. 2:**
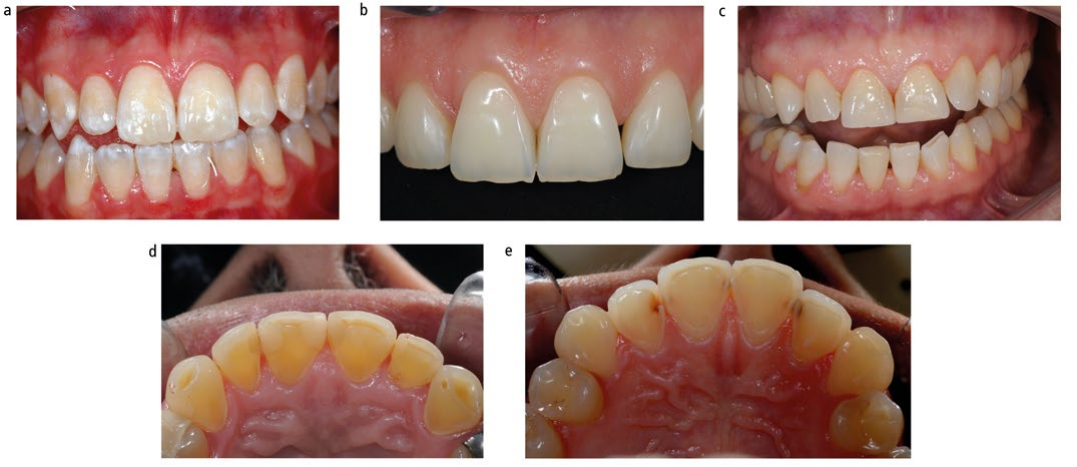
a) BEWE score 0 on anterior teeth showing unworn teeth. b) BEWE 1 showing a discrete area of wear on the UR1 on the buccal (facial surface) but no other signs of wear. c) BEWE 2 -shows less than 50% loss with signs of erosive tooth wear on the buccal (facial) surface but also some loss of the incisal edge. d) BEWE 3 greater than 50% of the surface affected. e) In this case the erosion has removed all of the palatal enamel giving a score 3

These are presented in Figure 2a.

#### BEWE Score 1

- First tooth wear signs: initial loss of surface texture (brightness loss, opaque surface or 'frosted glass' appearance) but with a discrete area on the buccal (facial) surface and minimal loss of the incisal edge.

This is presented in Figure 2b.

#### BEWE Score 2

- Distinct defect. Hard tissue loss less than 50% of the surface area. Dentine is often involved
- If there is loss of clinical crown height less than 50% from the buccal aspect, a score of 2 is given.

This is presented in Figure 2c.

#### BEWE Score 3

- Hard tissue loss signs with more than 50% of the surface area. Dentine is often involved but is not a prerequisite for a BEWE score of three
- For restored teeth, the tooth wear can only be scored provided that the size of the restoration does not exceed 50%.

This is presented in Figure 2d and 2e.

### Incisal edges of anterior teeth

**Fig. 3:**
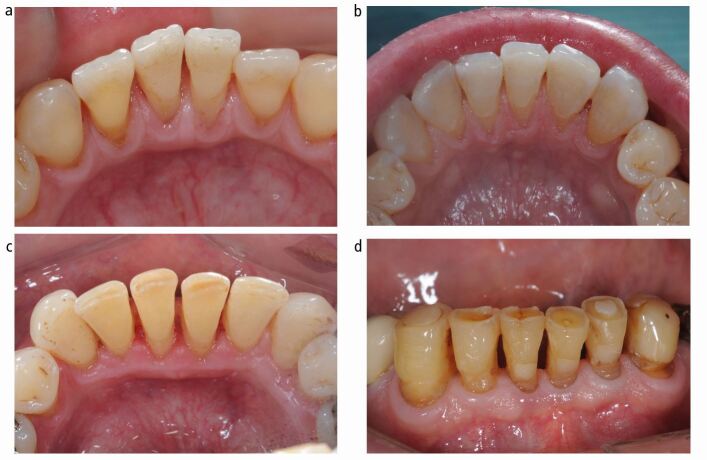
a) However slight wear is visible on the incisal surface of the canines so a sextant score of 1 is representative of wear in this sextant. b) the whole of the incisal edge of the two central incisors has wear and given a score 3 whereas on the canines the wear is less and would be a 2. The sextant score is 3. c) The wear has clearly involved the whole incisal edge and is given a 3. d) The wear is clearly seen on the incisal edge but there has also been some shortening of the teeth

Evaluating the wear along the incisal edges (Fig. 3a) can be challenging. However, the same criteria must be used as for other surfaces, even though this might result in a higher sextant score.

### Gingival recession

**Fig. 4:**
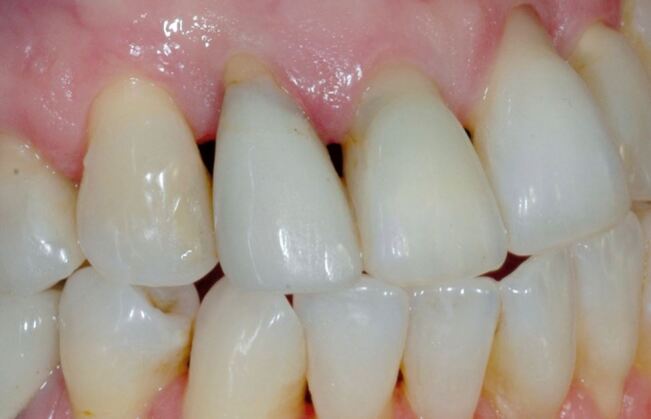
If a blunt end probe is run along the margin it is easier to determine as a normal feature

A differential diagnosis between erosive tooth wear with exposure of root and gingival recession (Fig. 4) is needed. The area can present a small depression or notch, anatomically normal. In case of doubts, a ball-ended probe (WHO) can be used to make a tactile assessment, comparing with neighbouring teeth. The apparent concavity around the gingival margin is not wear but the normal architecture of the tooth.

#### Crowns

**Fig. 5:**
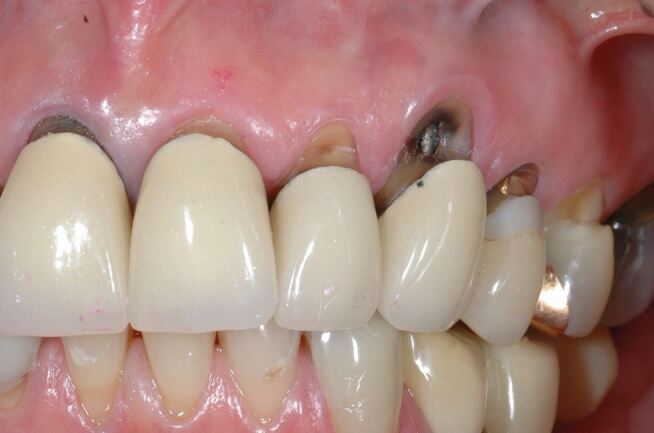
Even though tooth wear has produced the lesions associated with the crowns they cover more than 50% of the surface and therefore the sextant cannot be scored

When erosive tooth wear has been restored with crowns (Fig. 5), there may be a clinical suspicion that the reason is tooth wear, but if the restoration covers more than 50% then the surface cannot be scored.

#### Rotated teeth

In cases of tooth rotation, the surface facing the cheek would be buccal even if that would not be correct anatomically. The result of a rotation would not influence the sextant score.

#### Missing teeth

If a single tooth remains in a sextant, add the scoring to the adjacent sextant.

#### Differential diagnosis between erosive tooth wear and root/cervical caries lesion

- Colour assessment: root caries lesions appear brown-orange in colour. Non-carious wear will have the corresponding tooth structure colour. Compare the colour with the adjacent teeth
- Tactile sensation: carious lesions can present a friable surface. Non-carious wear will present a hard surface. Use the WHO probe in case of doubts and compare with neighbouring teeth
- Plaque assessment: plaque stagnation will be associated with a caries lesion
- If you detect a caries lesion associated with an erosive tooth wear lesion, consider the corresponding BEWE score for the affected surface, regardless of the caries lesion
- If still in doubt whether it is a caries lesion or an erosive tooth wear lesion, consider it as caries and register BEWE score 0.

**Fig. 6:**
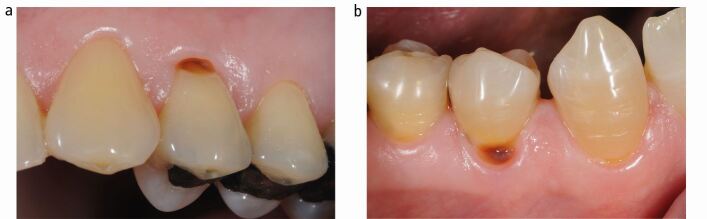
a) The lesion is carious and not erosive. b) Again a primarily carious lesion even though it appears to have some evidence of wear

The differential diagnosis between erosive tooth wear and root/cervical caries lesion is presented in Figs 6a and 6b.

## Conclusion

The BEWE has been shown to be a simple, validated tool to be used in primary dental care.^4^ This is a detailed paper explaining the BEWE; however, the most important aspect is to be reproducible for your own records and to screen for wear in everyday practice.
